# Challenges in Dietary Therapy in Pediatric Eosinophilic Esophagitis (EoE)—A Narrative Review

**DOI:** 10.3390/nu18010082

**Published:** 2025-12-26

**Authors:** A. Stochel-Gaudyn, A. Kozioł-Kozakowska, K. Kowalska-Duplaga

**Affiliations:** Department of Pediatrics, Gastroenterology and Nutrition, Jagiellonian University Medical College, 31-008 Cracow, Poland; agnieszka.koziol-kozakowska@uj.edu.pl (A.K.-K.); kinga.kowalska-duplaga@uj.edu.pl (K.K.-D.)

**Keywords:** eosinophilic esophagitis, dietary therapy, challenges, elimination diet

## Abstract

Eosinophilic esophagitis (EoE) is a chronic, allergic, immune-mediated inflammation of the esophagus caused by food antigens. The prevalence in pediatric population is approximately 34 to 57 cases per 100,000 children, with a male to female ration 3:1. This number may be underestimated due to diagnostic challenges and variety of clinical presentations in different age groups. Diagnosis of EoE requires histopathological assessment of esophageal biopsies retrieved during gastroscopy, with at least 15 eosinophils per high-power field (HPF) in the esophageal tissue being the cut off value. According to recommendations, treatment options of EoE include dietary interventions (elimination diets), medical treatment (inhibitors of proton pump, steroids, biologics), and in some cases surgical intervention (dilation). Dietary intervention, such as elimination diets, target the triggering factors of the disease and, if supervised by professional nutritionist, have the least systemic side effects. On the other hand, depending on the number of allergens eliminated from the pediatric patients’ diet, the quality of life both of the child and their caretakers may be compromised. Additional challenges such as nutritional risks, feeding disorders, financial burden, and social life impairment also have to be taken into consideration. On top of this, an effectiveness assessment of chosen therapy requires repeated endoscopic examination with several biopsies of the esophagus, further increasing diseases burden in EoE patients. Taking all of this factors into consideration, the main objective of this narrative review was to address challenges that pediatric patients with EoE on dietary treatment face with reference to current research and daily practice.

## 1. Introduction

Eosinophilic esophagitis (EoE) is a chronic, allergic, immune-mediated inflammation of the esophagus caused by food antigens. The prevalence in the pediatric population is approximately 34 to 57 cases per 100,000 children, with a male to female ration 3:1. This number may be underestimated due to diagnostic challenges and variety of clinical presentations in different age groups [1,2,3]. The symptoms of eosinophilic esophagitis (EoE) are highly varied and nonspecific, especially in the pediatric population. The diagnosis of EoE is a clinicopathologic one and requires the exclusion of other causes of eosinophilic inflammation of the esophagus [4,5].

Upper gastrointestinal endoscopy (EGD) with biopsies remains the gold standard for both the diagnosis and monitoring of EoE. It allows for the assessment of characteristic features resulting from chronic inflammation and tissue remodeling. An efficient diagnostic process is clinically important because prolonged duration of undiagnosed EoE increases the risk of progression to a fibro-stenotic form of the disease and the development of permanent complications [6].

Up to one-third of children with EoE may have a macroscopically normal-appearing esophagus. Endoscopic inflammatory features (such as exudates, edema, and furrows), although characteristic, can be subtle and easy to miss. Therefore, the endoscopic appearance of the esophagus cannot confirm or exclude the diagnosis of EoE [7]. The European Society of Pediatric Gastroenterology, Hepatology and Nutrition (ESPGHAN) recommends that esophageal biopsies be taken whenever EoE is being considered, regardless of the macroscopic appearance of the esophagus.

For the diagnosis and monitoring of EoE in children, it is recommended to obtain at least six biopsy samples from the upper, middle, and lower levels of the esophagus (at least two from each level), targeting visible lesions whenever possible [8]. The need to obtain multiple biopsies from different levels of the esophagus arises from the focal nature of inflammation in EoE, meaning that the distribution of eosinophils in the esophagus can be patchy [9]. It has been shown that taking biopsies only from the distal esophagus can result in a false-negative finding. Histologic diagnosis is based on the peak eosinophil count (PEC) of at least 15 eosinophils per high-power field (eos/HPF). It is emphasized that proper interpretation of histopathologic findings also requires standardization. The size of the high-power field (HPF) varies depending on the technical specifications of the microscope. Therefore, it is recommended to use a standardized tool for reporting eosinophil density, including converting eos/HPF values to eos/mm^2^ or to a standardized HPF size (e.g., 0.27 mm^2^) to enable comparability of results [10].

According to recommendations, treatment options for EoE include dietary interventions (elimination diets), medical treatment (inhibitors of proton pump, swallowed topical steroids, biologics), and, in some cases, surgical intervention (dilation) [11].

Dietary therapy remains the only treatment that directly targets the underlying cause of EoE [12]. Current management relies on three main nutritional strategies: elemental diets, empiric elimination diets, and elimination guided by allergy testing [13,14]. In recent years, practice has shifted from highly restrictive approaches toward more streamlined and practical options, aiming to maintain efficacy while improving adherence and quality of life, particularly in children. Understanding these evolving dietary pathways is therefore essential when considering therapeutic choices in pediatric EoE [8,15].

The main objective of this narrative review was to address challenges that pediatric patients with EoE on dietary treatment face, with reference to the research available in the literature and daily practice.

## 2. Materials and Methods

This article is a narrative review aimed at summarizing current evidence regarding dietary therapy in pediatric eosinophilic esophagitis (EoE), with particular focus on the clinical, nutritional, psychosocial, and economic challenges associated with dietary management.

A literature search was conducted in the databases PubMed, Scopus, Web of Science, and Google Scholar. Suitable entries published between 2013 and 2025 were screened, reflecting the rapid evolution of diagnostic criteria and dietary treatment strategies over the past decade. To ensure comprehensive coverage of the topic, clinical practice guidelines, expert consensus statements, systematic reviews, meta-analysis, and observational studies were included.

Studies were excluded if they focused exclusively on adult populations, examined pharmacological or endoscopic treatment without dietary intervention, consisted only of conference abstracts without full text, or lacked sufficient methodological quality based on authors’ judgment.

Due to the narrative nature of this review, quantitative meta-analysis and formal risk-of-bias assessment were not conducted. Instead, emphasis was placed on qualitative synthesis and comparative interpretation of available evidence, highlighting gaps in knowledge, variability in treatment protocols, and practical difficulties encountered in pediatric dietary management of EoE.

## 3. Elemental Diets in Pediatric EoE

Elemental diets consist of exclusive intake of amino acid-based formulas and represent the most effective dietary therapy for EoE. Cumulative data, including meta-analysis, show histologic remission rates exceeding 90% in both children and adults, with improvement occurring rapidly, sometimes within two weeks in adult patients [14]. Despite this high level of efficacy, elemental therapy is rarely used as an initial approach in routine clinical practice [8,15].

Several factors limit its feasibility, including poor palatability leading to reduced adherence, high cost and inconsistent coverage, and the need for a prolonged food reintroduction period requiring multiple endoscopic assessments. These challenges are associated with significant treatment burden, including social restrictions and increased school or work absenteeism. For this reason, elemental diets are generally reserved for patients who do not respond to empiric elimination or pharmacologic therapy, for young children in whom formula feeding is more acceptable, and for selected individuals receiving enteral nutrition via gastrostomy [16,17].

In infants and young children, oral motor skills should be maintained through age-appropriate amino acid-based semisolid preparations and transition to solid foods once remission is achieved. Exclusive elemental therapy is typically continued for at least four weeks before repeat endoscopy is performed to confirm histologic response [15].

## 4. Empiric Elimination Diets in Pediatric EoE

Empiric elimination diets are currently the most practical and widely supported dietary strategy for EoE, as they remove food groups known to commonly trigger the disease without relying on allergy testing. The classic six-food elimination diet (SFED) excluding cow’s milk, wheat/gluten, egg, soy/legumes, nuts, and fish/seafood has consistently demonstrated clinical and histologic remission in roughly 70–75% of patients across both pediatric and adult studies, including meta-analysis [8,14,18]. Local adaptation of excluded foods is sometimes applied to reflect regional dietary patterns, helping to optimize response.

Despite its effectiveness, SFED is associated with significant challenges, including reduced quality of life, high dietary burden, the need for intensive nutritional guidance, and prolonged reintroduction periods requiring multiple endoscopies. These limitations have led to the development of less restrictive approaches. A four-food elimination diet (FFED), typically removing cow’s milk, wheat, egg, and soy/legumes, achieves remission in approximately 50–65% of children and adults, while identifying milk and egg as the most frequent triggers upon reintroduction [19]. Building on this concept, a step-up protocol (2-4-6 FED) begins with a two-food elimination (TFED), dairy and gluten-containing cereals and progresses to FFED or SFED only in non-responders. This staged method has been shown to reduce the number of endoscopies and shorten the diagnostic process, with nearly half of patients responding to the initial two-food phase [8].

Given that cow’s milk is the most common single trigger in pediatric EoE, isolated milk elimination has also been evaluated as a first-line option. Studies in children report histologic remission rates of around 50–65%, with comparable symptom improvement to more restrictive diets and better psychosocial outcomes. Recent adult data similarly suggest that starting with milk elimination may achieve short-term results equivalent to SFED while imposing fewer dietary burdens [20].

The distinction between eliminating wheat versus all gluten-containing cereals can create confusion in dietary management of EoE [21]. Current evidence indicates that wheat is the more relevant trigger, and routine exclusion of barley and rye is not recommended. ESPGHAN therefore advises maintaining standard protocols based on wheat elimination rather than extending to full gluten avoidance, as the theoretical risk of cross-reactivity has not been supported by data. However, because gluten-free products are often more clearly labeled and widely available, some families may find it easier to implement wheat elimination using gluten-free options, despite this not requiring complete gluten restriction [8].

A systematic review of 34 studies published between 2005 and 2022 showed that within SFED and FFED interventions, 17 studies excluded wheat only, while just 4 studies removed all gluten-containing cereals, and no studies specifically evaluated gluten-focused two-food elimination in children. Given the limited evidence and study heterogeneity, wheat elimination remains the preferred approach, and broader gluten restriction should be reserved for future investigation [22].

Overall, the shift toward less restrictive empiric strategies reflects the need to balance efficacy with sustainability. In children, minimizing unnecessary food exclusions is crucial to protect growth, prevent nutrient deficiencies, and reduce the risk of disordered eating [8].

## 5. Elimination Diets Guided by Allergy Testing in Pediatric EoE

Elimination diets guided by allergy testing aim to identify causative foods based on immune-mediated mechanisms, such as food-specific IgE or cell-mediated pathways, rather than empirically excluding the most common allergens. In theory, this approach offers a more targeted strategy than empiric elimination, potentially reducing dietary restrictions [15].

However, the evidence base for allergy testing-guided diets is limited. Most studies are single-arm and observational, reporting highly variable outcomes. Pooled data suggest that nearly half of all patients fail to achieve histologic remission (<15 eosinophils/hpf) when diets are based solely on allergy testing. Differences in testing techniques, including skin prick tests, serum-specific IgE, and patch testing, alone or in combination, contribute to inconsistent results. Sensitivity analyses have not demonstrated clear superiority of any specific testing method, and the role of aeroallergen testing remains under investigation [8,23].

Additional challenges include long-term adherence to restrictive diets, uncertainty regarding the development of new IgE-mediated food allergies upon reintroduction, and limited correlation between symptoms, endoscopic findings, and histologic disease activity. As with other elimination strategies, repeated endoscopies are required to confirm remission and identify triggers, imposing a significant burden on children and families [23,24].

In practice, allergy testing-guided elimination diets are generally considered secondary options, often reserved for cases in which empiric or step-up approaches are not feasible or when testing may help clarify suspected immune-mediated triggers. Current evidence suggests that, while conceptually appealing, this strategy does not reliably predict food triggers in pediatric EoE, highlighting the need for further research to improve accuracy and clinical applicability [24]. The comparative characteristics, efficacy, and limitations of the available dietary interventions are shown in Table 1.

## 6. Disease Monitoring and Management (Burden of Frequent Gastroscopies)

Normalization of eosinophils in the mucosa is one of the main goals of EoE treatment, alongside improvement of symptoms and endoscopic appearance. Dietary therapy, including elimination diets (e.g., four-food elimination diet, FFED) and elemental diets (amino acid-based formula, AAF), requires a strict schedule of endoscopic evaluation during the remission induction phase, which can be a serious limitation for this therapeutic option [25].

ESPGHAN recommends a minimum of 8 to 12 weeks of elimination diet therapy, topical steroids or PPIs before reassessment by endoscopy. After this period, endoscopic re-evaluation with biopsies should be performed to verify whether histologic remission has been achieved [8]. In the case of elemental diet therapy (AAF, containing only amino acids), endoscopic re-evaluation is recommended no earlier than 4 weeks after induction. Definition of histologic remission remains controversial and varies between studies (e.g., <6 eos/HPF in randomized controlled trials versus <15 eos/HPF in many observational studies), as summarized by Van Rhijn et al. [24] and recent ESPGHAN guidance [8].

Endoscopy also remains crucial during the maintenance phase after remission has been achieved with dietary or pharmacologic therapy. In the event of clinical relapse during maintenance, the ESPGHAN EGID WG (Eosinophilic Gastrointestinal Disease Working Group) recommends endoscopic and histologic evaluation. In patients with stable clinical remission, endoscopic and histologic re-evaluation is recommended after 1–3 years of the maintenance phase [8].

In summary, the need for endoscopic monitoring is intrinsically linked to both dietary and pharmacologic treatment of EoE. Endoscopy is the only tool that allows for an objective assessment of histologic remission. Therefore, endoscopic surveillance is also fundamentally important for monitoring esophageal status and identifying relapses or the development of structural changes, regardless of clinical symptoms. Taking this into consideration, frequent endoscopies, especially shortly after EoE diagnosis, pose an important challenge in pediatric patients since they often require anesthesia and hospitalization.

## 7. Endoscopy as a Therapeutic Method in Pediatric EoE

Therapeutic endoscopy in EoE is most commonly indicated for the removal of impacted food boluses or for the dilation of strictures in patients with fibro-stenotic changes. In the former case, the procedure is urgent and unplanned, escalating patients stress.

Therapeutic dilation, on the other hand, should be performed in conjunction with standard induction and maintenance therapy in a scheduled timeframe. Dilation is generally considered when the esophageal diameter is too narrow, with the therapeutic goal of achieving a lumen of 16–17 mm [26]. While dilation alleviates dysphagia, it does not address the underlying inflammatory process or induce histologic remission like dietary therapy.

Prior to planned dilation, especially in cases of severe strictures, a barium swallow (BS) is recommended to assess the length, severity, and location of the stricture, which is often difficult to evaluate using endoscopy alone. Barium imaging is also useful for assessing esophageal anatomy when severe strictures prevent even a narrow endoscope from passing. On the other hand, BS exposes the patient to X-rays and increases the amount of medical procedures, further increasing the disease burden.

## 8. Clinical and Practical Considerations for Pediatric Endoscopy in EoE

The need for repeated endoscopic evaluation in the treatment and monitoring of EoE presents a major challenge in the management of pediatric patients, as standard endoscopy in children and adolescents is typically performed under general anesthesia or sedation. Thus, the main risk associated with frequent procedures is the potential effect of repeated exposure to esthetic agents at a young age. This may include respiratory complications, drops in blood pressure, nausea, vomiting, and, very rarely, serious adverse events. The risk increases in children with comorbidities (e.g., asthma, heart defects, obesity). Upper gastrointestinal endoscopy, especially when interventional procedures are involved, carries a small but real risk of perforation, bleeding, and dental injury. In children with EoE, the risk of perforation is higher particularly in the presence of strictures and the need for esophageal dilation, although even then it is described as very low [8].

Psychological burden of frequent endoscopies is also significant. In quality-of-life studies, parents and patients with EoE identify the number of endoscopies as an important source of stress, anxiety, and school/work absenteeism. It is also important to recognize that each endoscopy entails hospitalization or a day-stay visit, travel to a gastroenterology center, and the need to arrange child-care, which, when several procedures per year are required, places a substantial financial burden on the family [27].

To reduce the number of follow-up endoscopies, the ESPGHAN guidelines recommend strategies such as a step-up dietary approach, which allows for fewer surveillance endoscopies, and the continuation of maintenance therapy to prevent relapses that would necessitate additional biopsies [8].

To avoid frequent sedation during EoE monitoring, minimally invasive methods are being investigated, including unsedated transnasal endoscopy (TNE), often combined with virtual reality goggles. However, these techniques still require further validation and acceptance, particularly in pediatric practice.

## 9. Other Diagnostic Methods in EoE

Transnasal esophagoscopy (TNE) is a safe and less costly alternative to conventional esophagogastroduodenoscopy performed under general anesthesia. It could therefore be considered as an alternative to standard unsedated endoscopy, particularly for monitoring therapy, such as dietary therapy, in children. TNE is performed under topical anesthesia, using audio or visual distraction techniques to reduce patient discomfort. Studies have shown that TNE is safe, allows for adequate biopsy sampling, is less expensive, and requires less procedure time compared to standard endoscopy. However, its use still requires further validation in larger pediatric cohorts [11].

Currently, none of the nonendoscopic methods for obtaining esophageal samples or biomarkers have been validated in the pediatric population. Among the most promising approaches are the Esophageal String Test (EST), Cytosponge^®^, and Blind Esophageal Brushing (BEB). EST involves inserting a thin string with a capsule at its end through the nose, which remains in the esophagus overnight and is then removed for analysis of eosinophil-related biomarkers, such as eosinophil cationic protein (ECP) and eosinophil-derived neurotoxin (EDN), from material washed off its surface. Cytosponge^®^ is a noninvasive technique in which a capsule containing a retractable sponge is swallowed; once the capsule dissolves, the sponge expands, collects epithelial cells during withdrawal, and the material is analyzed for eosinophils and eosinophil-related biomarkers, including pan-eosinophil markers like MPO and ECP. BEB involves inserting a cytology brush through a nasogastric tube without endoscopic visualization to obtain esophageal epithelial samples for biomarker analysis, such as EDN. While these methods show promise, they require further validation in pediatric populations, both in terms of efficacy and tolerability, as procedures considered minimally invasive in adults may provoke significant anxiety and discomfort in children [6]. Other promising techniques include probe-assisted confocal endomicroscopy, magnifying endoscopy with narrow-band imaging, and endoscopic esophageal ultrasound; however, these methods still require further investigation.

## 10. Feeding Difficulties

Feeding difficulties (FD) is a broad term that encompass a wide spectrum of clinical presentations such as selective or restrictive eating, neophobia, food avoidance, disruptive meal behavior or experiencing discomfort/pain while eating [28]. The severity of FD may vary from mild to severe, including conditions such as Avoidant Restrictive Food Intake Disorder (ARFID), and impact child’s proper physical and neurological development. The overall prevalence of FD in pediatric population oscillates between 25 and 45%; this wide range can be explained by heterogeneity of FD definition. In pediatric population with chronic diseases, FD reached 40–70%, including in EoE pediatric patients where FD varies between 13 and 75%, depending on the definition and diagnostic tools [29,30].

The diagnosing and managing of feeding difficulties in pediatric patients with EoE is especially challenging. As EoE is a chronic inflammation of the esophagus, it leads to dysmotility or even narrowing, presenting clinically as dysphagia, chest pain, nausea, vomiting or food impaction, which usually cause FD. The age of FD symptoms presentation may postpone EoE diagnosis since it is well documented in the literature that even healthy children experience food avoidance or neophobia at some point of their development [31]. Children with EoE adapt to esophagus dysmotility by increasing fluids intake with meals, eating small portions, prolonging chewing and mealtimes or avoiding difficult textures [32]. The diagnosis of FD may precede EoE diagnosis by up to 3 years, being misinterpreted as age-related food avoidance and postponing adequate treatment. This is why it is crucial to take EoE into consideration for differential diagnosis of FD [33].

On the one hand, feeding difficulties may be a clinical symptom of EoE; on the other hand, they may be a consequence of dietary therapy, which is one of the treatment options for this condition. Dietary therapy of EoE consists of elimination diets. Depending on the number of triggering allergens, the diets may be very restrictive, influencing the meals’ taste and texture. Exposure to different food textures is a key part in developing proper oral motor skills and sensory acceptance in young children [34]. In older children, elimination diets may cause excessive stress and anxiety during meal times, leading to food avoidance [35]. In all age groups, restrictive diets can cause nutritional deficiencies, influencing the child’s proper growth and development [36].

## 11. Quality of Life

Quality of life (QoL) of pediatric patients with EoE on dietary treatment can be influenced in two ways, both positively by relieving disease symptoms and negatively due to food restrictions leading to social life impairment or such burdens as emotional, physical or financial ones.

Several studies addressed the QoL in reference to the type of elimination diet in EoE patients. Restrictive diets are associated with higher reemission rates but lower QoL, thus leading to poor compliance [37,38]. Besides social life impairment and financial burden, patients on restrictive diets usually have more refractory diseases and may require more frequent endoscopies in order to monitor disease activity, further decreasing the QoL.

Available data also report compromised family QoL among these patients. Daily activity is limited due to the time necessary to purchase proper groceries and prepare meals compared to families with children suffering from another chronic condition [39]. In addition, lack of proper education or support with managing elimination diet can lead to anxiety and exacerbate disease burden [40]

On the other hand, good symptom management, especially in patients with identified food triggers, can improve QoL. Studies suggest a step-up dietary strategy, which comprises starting an elimination diet with one or two allergens, and only escalating elimination further products when necessary, improving adherence to the restriction diet and QoL of the patient and their family [38]. Thus, balancing effective symptom management and implementing the least restrictive diet in order to optimize QoL is crucial in pediatric patients with EoE.

## 12. Social Life Restrictions

Social and family life often revolve around food or activities where food or snacks play an important role, such as birthday parties, school events, and family and holiday gatherings. Since dietary therapy in pediatric EoE requires elimination diets, it can have a vast impact on social activities. Additionally, anxiety or embarrassment related to clinical symptoms of EoE such as slow eating, regurgitation, necessity of increase fluid intake after eating or dysphagia may further impact social life [41].

In small children, the social life restrictions instead tend to impact the parents and caretakers. Family gatherings and outings to restaurants may be challenging. Usually, parents have to plan ahead where they can eat while dining out, or have to plan which meals or snacks to bring with themselves. This can lead to stress and additional financial burden, impacting the decision to go out.

In preschool and school children, elimination diets may lead to social distancing and avoidance of gatherings. The necessity to decline snacks or eating special meals can cause peer rejection and stigma. Furthermore, the feeling of being different can cause mood decline, stress or even depression, as well as social distancing or skipping school and extracurricular activities [40].

Based on recent studies, social limitations may influence as much as two-thirds of EoE pediatric patients. A total of 69% children with EoE report some difficulties in their social life, whereas 34% admit to a moderate or severe impact that the disease has on their social life [41].

## 13. Financial Burden

Financial burden in dietary therapy of EoE is considerable, especially in pediatric patients where this kind of therapy is often the most common one. The amount of expenses will depend on the type of diet, whether it is formula based or requires specific allergens elimination. The costs often exceed those of medications or medical procedures. The patients’ involvement in costs will vary in reference to where they live and their insurance [42].

In young children, amino acid-based formulas are often chosen for EoE dietary treatment. They are extremely expensive, and the insurance rarely covers the total cost, thus forcing the parents or caretakers to partake in this substantial expense out of their own pocket.

In older children, the dietary treatment of choice is elimination diets, which often remove many major food groups or common products, such us dairy, wheat, soy or egg. Such diets require the purchase of allergen-free or specialized food products, which usually are expensive. On top of that, in order to balance nutritional requirements, supplements of vitamins and micronutrients are often needed, which increases the expenses even more [43].

Adequate dietary treatment in EoE should be supervised by a professional and experienced clinical dietitian. In order to ensure a child’s proper development and growth, consultations with a dietitian should be frequent, especially in young children. Unfortunately, not all countries and insurance companies provide free dietary consultations, further increasing out of pocket expenses [44].

Annual dietary therapy of EoE cost can vary from hundreds to several thousands of dollars per family depending on the type of diet and age and size of the child. This financial burden can lead to lack of compliance and in effect to poor disease control [45,46].

## 14. Conclusions

Each dietary treatment strategy in pediatric EoE carries specific benefits and limitations: elemental diets offer the highest efficacy but are often impractical; empiric elimination diets balance effectiveness with feasibility; and allergy testing-guided diets have shown inconsistent results and are generally reserved for selected cases [8,12,13].

It is important to emphasize that initial elimination diets are not intended to be permanent. The primary goal is to induce remission and then systematically reintroduce foods to identify the true triggers. Ultimately, lifelong dietary management typically involves the elimination of one to three key culprit foods, most commonly dairy, gluten-containing cereals, and eggs [15].

A step-up approach, beginning with the least restrictive diet and escalating only if needed, is increasingly favored in pediatric practice. This strategy minimizes nutritional risk, supports adherence, and reduces the endoscopies needed as well as the psychosocial impact on children and families. When dietary therapy is chosen, a step-up approach supported by experienced dietetic supervision remains essential to maintain nutritional adequacy and adherence. The importance of multidisciplinary approach, including gastroenterologists, allergists, dietitians, and psychologists, is crucial. Effective implementation requires careful planning, thorough patient and caregiver education, and ongoing monitoring to ensure both disease control and maintenance of growth, nutrition, and quality of life [8].

## Figures and Tables

**Table 1:** Characteristics and clinical comparison of dietary therapies used in pediatric eosinophilic esophagitis (EoE).

Diet Type	Histologic Remission (%)	Advantages	Limitations
Elemental diet (AAF)	>90%	Highest response rate; antigen-free formulation	High cost; low palatability; potential feeding aversion; limited social acceptance
SFED (Six-Food Elimination)	~70–75%	High efficacy; well-documented outcomes	Most restrictive; nutritional risk; multiple endoscopies required
FFED (Four-Food Elimination)	~50–65%	Balanced restriction vs. SFED; good clinical response	May require escalation to SFED or elemental diet
TFED (Two-Food Elimination)	~45–55%	Best adherence; improved QoL and feasibility	Lower remission vs. FFED/SFED; step-up strategy often necessary
Allergy-test-guided elimination	~30–50%	Individualized approach possible	Low predictive accuracy of testing; inconsistent efficacy

Values represent approximate ranges reported across pediatric cohorts. TFED typically eliminates cow’s milk and wheat as first-line allergens; FFED excludes milk, wheat, egg, soy; SFED additionally excludes nuts + fish/seafood. Elemental diet reserved for escalation or severe disease.

## Data Availability

No new data were created or analyzed in this study.

## Abbreviations

The following abbreviations are used in this manuscript:

EoE	Eosinophilic esophagitis
HPF	High-power field
EGD	Upper gastrointestinal endoscopy
ESPGHAN	European Society of Pediatric Gastroenterology, Hepatology and Nutrition
SFED	Six-food elimination diet
FFED	Four-food elimination diet
TFED	Two-food elimination
FD	Feeding difficulties
QoL	Quality of life
TNE	Transnasal esophagoscopy
EST	Esophageal String Test
BEB	Blind Esophageal Brushing
ECP	Eosinophil cationic protein
EDN	Eosinophil-derived neurotoxin
